# CMCSMA-Citric Acid Hydrogel-Coated Pancreatic Duct Stent Used for Pancreatic Calculi

**DOI:** 10.3390/gels11080651

**Published:** 2025-08-16

**Authors:** Jing Li, Jiahao Yang, Shige Wang

**Affiliations:** 1 School of Materials and Chemistry, University of Shanghai for Science and Technology, No. 334 Jungong Road, Shanghai 200093, China; lijing@usst.edu.cn (J.L.); y2952847512@163.com (J.Y.); 2 Public Experiment Center, University of Shanghai for Science and Technology, No. 334 Jungong Road, Shanghai 200093, China

**Keywords:** pancreatic calculi, hydrogel, carboxymethyl chitosan methacrylate, citric acid, drug-eluting stent

## Abstract

Pancreatic calculi, a common complication of chronic pancreatitis, significantly contribute to ductal obstruction, increased intraductal pressure, and debilitating abdominal pain. Although endoscopic pancreatic duct stenting alleviates ductal stenosis, conventional stents lack litholytic functionality, limiting their therapeutic efficacy. To address this challenge, we developed a drug-eluting pancreatic duct stent coated with a carboxymethyl chitosan methacrylate (CMCSMA)-based hydrogel utilizing 50% *w*/*v* citric acid (CA) as a litholytic agent. Polydopamine (PDA) interlayer was employed to enhance interfacial adhesion between the hydrogel and the stent surface. The CMCSMA hydrogel exhibited favorable physicochemical properties, including rapid gelation, excellent compressive strength (229.2 ± 14.8 kPa), hemocompatibility, and cytocompatibility. In vitro release studies revealed sustained CA release, achieving 66.3% cumulative release within 72 h. The hydrogel-coated stent demonstrated superior litholytic activity, dissolving over 90% of pancreatic calculi within 24 h. These results underscore the potential of CMCSMA-CA hydrogel-coated stents as a biocompatible and effective local drug delivery platform for targeted pancreatic duct litholysis.

## 1. Introduction

Pancreatic calculi, which develop within the pancreatic ductal system, are predominantly associated with chronic pancreatitis, long-term alcohol abuse, hereditary disorders, and obstructive pancreatic pathologies. Composed predominantly (~95%) of inorganic calcium salts, pancreatic calculi mainly consist of calcium carbonate and calcium phosphate [1,2]. Citric acid (CA), tartaric acid, and other organic acids can effectively dissolve calculi by providing protons and chelating Ca^2+^ [3,4,5]. CA can be utilized to treat protein blockage and calcification in the pancreas and has demonstrated considerable therapeutic effects [6]. Additionally, CA can dissolve pancreatic calculi and alleviate abdominal pain through oral administration or continuous infusion at the sites of the calculi [7]. However, the long-term oral administration of CA or citrate can cause side effects such as induced gastritis, gastric ulcers, gastric perforation, or stomach bleeding. Furthermore, continuous infusion via surgical procedures is time-consuming and brings pain and inconvenience to patients, failing to address the issue of postoperative calculi recurrence [6,7].

Current guidelines recommend a spectrum of endoscopic and surgical techniques for pancreatic calculi, including lithotripsy, stent placement, and ductal decompression [8,9]. With developments in pancreatoscopy, calculi can now be treated with intraductal lithotripsy, using either electrohydraulic energy or a holmium laser [10]. However, endoscopic techniques are not well suited for the management of large or hard pancreatic calculi, particularly those located in anatomically challenging regions [11]. Endoscopic retrograde cholangiopancreatography (ERCP), when combined with sphincterotomy, stenting, and extracorporeal shock wave lithotripsy (ESWL), can achieve effective pancreatic duct clearance [12,13]. However, this approach still faces challenges, including incomplete calculus removal and a high rate of postoperative recurrence.

Endoscopic pancreatic duct stents, including plastic (polyethylene) stents and fully coated self-expandable metal stents, can be implanted into the pancreatic duct via endoscopic procedures [14,15,16]. These stents effectively relieve pancreatic duct stenosis by gradually dilating and supporting the duct, thereby restoring physiological drainage [17,18]. However, pancreatic duct stents alone lack litholytic properties and are prone to stent obstruction and restenosis. The drug-eluting stent for pancreatic duct litholysis represents an innovative solution [19,20], consisting of three key components: a drug-carrying matrix material, a litholytic drug, and a pancreatic duct stent. Unlike traditional bare pancreatic duct stents, this combination of mechanical support and pharmacological action offers a promising strategy for improving outcomes in pancreatic duct disorders [19]. The low surface energy and non-reactive nature of conventional polyethylene stent polymers require plasma activation or chemical grafting to achieve adequate coating adhesion, with recent advances focusing on mussel-inspired polydopamine (PDA) interfaces for sustained drug delivery. PDA exhibits excellent biocompatibility and robust adhesion properties, making it widely employed as a polymeric coating for enhancing surface modifications of materials [21,22,23,24]. After PDA modification, the interfacial interaction between the stent surface and the hydrogel can be significantly improved, thereby effectively preventing hydrogel delamination during specific processes. In addition, the use of drug-eluting stents can eliminate the side effects caused by oral administration of drugs and prevent postoperative recurrence of pancreatic calculi [7].

Hydrogels represent an ideal drug delivery platform, demonstrating broad applicability in biomedical fields, and have been used as scaffolds for tissue engineering or as delivery carriers for therapeutic agents and cells [25,26,27]. Chitosan, a naturally occurring cationic polysaccharide derived from chitin, has emerged as an attractive biomaterial for drug delivery applications due to its excellent biocompatibility, biodegradability, and ready availability. However, the poor solubility of chitosan in neutral and alkaline aqueous media (pH ≥ 6.5) significantly restricts its pharmaceutical and biomedical applications. To overcome this limitation, chitosan can be chemically modified through carboxymethylation to produce carboxymethyl chitosan (CMCS). After carboxymethylation modification, CMCS has overcome the three major bottlenecks of solubility, bio-compatibility, and functional diversity while retaining the biological activity of CS, and is particularly suitable for drug delivery systems requiring pH stability and long-term implantable biomaterials [28]. CMCS possesses excellent biocompatibility, biodegradability, and antibacterial activity and physicochemical tunability, rendering it a highly suitable material for hydrogel fabrication [29,30,31]. CMCS-based hydrogels have received considerable attention for their ability to encapsulate a wide range of bioactive agents, including anticancer drugs, anti-inflammatory compounds, and therapeutic proteins, owing to their favorable controlled release properties [28,32,33]. The methacrylation of CMCS introduces photosensitive vinyl groups that participate in radical polymerization when exposed to UV light, transforming the aqueous CMCS methacrylate (CMCSMA) solution into a crosslinked CMCSMA hydrogel network within seconds. Nevertheless, the development of CMCS-based hydrogel drug-eluting stents for pancreatic duct litholysis remains unexplored. Compared with GelMA hydrogel [20], CMCSMA hydrogel has lower mechanical strength and is more flexible. Moreover, its degradation rate is faster, making it more suitable for the preparation of drug-eluting stents for pancreatic duct calculi, thereby avoiding the problem of secondary removal.

In this study, we developed a CMCSMA hydrogel and tried to fabricate a novel drug-eluting stent for the potential application in the interventional treatment of pancreatic duct calculi clearance. The stent consists of the plastic pancreatic stent and the CMCSMA hydrogel coating loaded with CA. In vitro drug release and litholysis experiments were carried out to assess the drug release kinetics and calculi-dissolving efficacy of the drug-eluting stent. The in vitro swelling and degradation of CMCSMA hydrogel coating was used to evaluate whether the hydrogel-coated stent is suitable for implanting into the pancreatic duct. The CMCSMA hydrogel-coated drug-eluting stent demonstrated superior performance in the in vitro swelling and degradation section.

## 2. Results and Discussion

### 2.1. Characterization of CMCSMA

**Figure 1:**
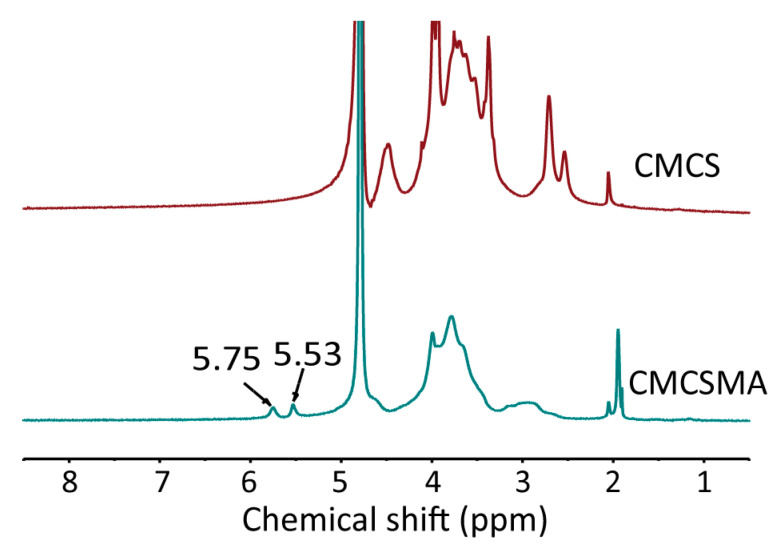
The ^1^H spectra of CMCS and CMCSMA [36].

In the ^1^H NMR spectrum of CMCSMA, the characteristic peaks observed at 5.75 ppm and 5.53 ppm correspond to the vinyl protons of the methacrylate groups, demonstrating the successful grafting of methacrylate groups onto the CMCS molecular chains. These distinct chemical shifts provide clear evidence for the introduction of a carbon/carbon double bond and confirm the successful synthesis of CMCSMA (Figure 1). The coupled doublets at the chemical shifts of 5.75 ppm and 5.53 ppm are assigned to the vinyl groups of MA (methacrylic anhydride), and the singlet at 1.8–2.0 ppm is due to the methyl group of the methacrylate units, which confirmed the methacryloyl functionalization of CMCS [34]. The quantitative analysis of the peak integration areas in the ^1^H NMR spectrum allowed for the calculation of the grafting degree of methacrylate groups, which was determined to be 13.5% according to a previous study [35].

### 2.2. PDA Modification and In Situ Synthesis of CMCSMA-CA Hydrogel on Pancreatic Duct Stent

**Figure 2:**
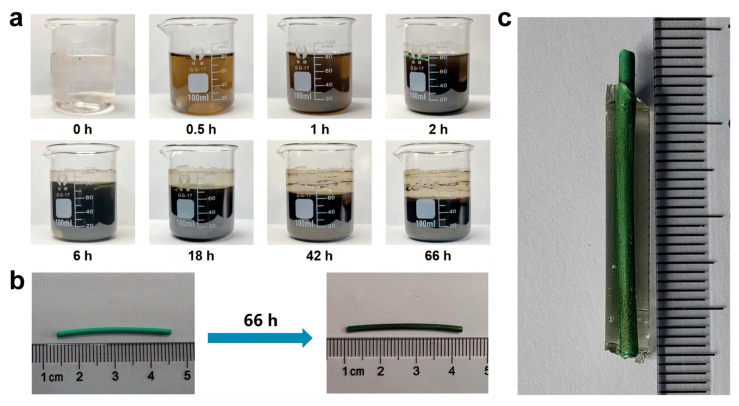
(**a**) Macroscopic images illustrating the color evolution of the solution during the oxidative self-polymerization of dopamine to form PDA; (**b**) comparison of an unmodified pancreatic duct stent and a PDA-modified pancreatic duct stent; (**c**) a PDA-modified pancreatic duct stent coated with CMCSMA-CA hydrogel.

Under aerobic and alkaline conditions, dopamine can undergo oxidation by atmospheric oxygen and self-polymerize to form PDA [37,38]. PDA, owing to its remarkable adhesive properties and excellent biocompatibility, has been extensively employed as a biomedical surface coating material. In the present study, PDA was generated in situ on the surface of a plastic pancreatic duct stent to improve its surface adhesion characteristics. The progression of dopamine oxidation was visually monitored by capturing images of the reaction solution at predetermined time intervals (0, 0.5, 1, 2, 6, 18, 42, and 66 h) using a digital camera (Figure 2a). As illustrated in Figure 2a, the solution color gradually changed from transparent to brown and ultimately to black, indicating the oxidative self-polymerization of dopamine into PDA. Concurrently, the surface color of the plastic stent changed from green to black (Figure 2b), further confirming the successful deposition of PDA on the stent surface. Following PDA surface functionalization, a CMCSMA-CA hydrogel was photopolymerized in situ on the stent substrate to form a drug-eluting hydrogel coating. As shown in Figure 2c, the hydrogel was successfully coated onto the PDA-modified stent.

### 2.3. Characterization of Hydrogels

**Figure 3:**
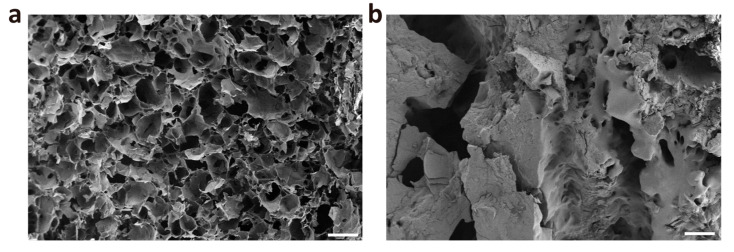
(**a**) SEM image of freeze-dried CMCSMA hydrogel; (**b**) SEM image of freeze-dried CMCSMA-CA hydrogel (scale: 200 μm).

The CMCSMA and CMCSMA-CA hydrogel samples were freeze-dried, and their microstructures were characterized using SEM. As shown in Figure 3a, the CMCSMA hydrogel exhibits a highly porous mesh-like structure with interconnected pores, which is advantageous for the loading of litholytic drugs. In contrast, after the loading of CA, the internal porosity of the CMCSMA-CA hydrogel is significantly reduced, with a substantial portion of the pore space occupied by CA (Figure 3b). These observations demonstrate that CMCS-based hydrogels possess the capacity to accommodate large quantities of water-soluble drugs, making them suitable for drug delivery applications.

### 2.4. Rheological and Mechanical Properties of Hydrogel Coating

**Figure 4:**
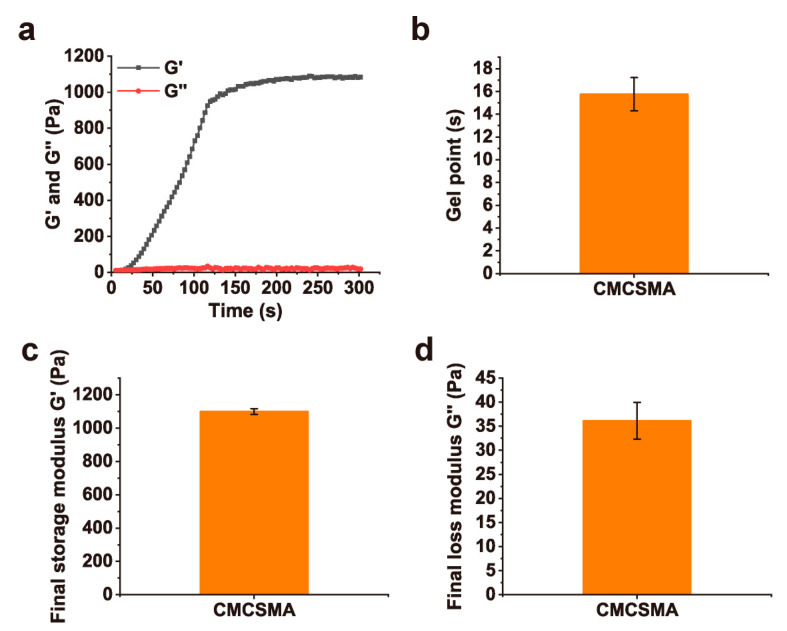
(**a**) Time-sweep oscillatory test results of CMCSMA hydrogel; (**b**) gelation time of CMCSMA hydrogel; (**c**) final G′ value of the CMCSMA hydrogel; (**d**) final G″ value of the CMCSMA hydrogel.

To minimize the potential influence of the stent on the rheological and mechanical properties, and considering that CA does not participate in the hydrogel’s crosslinking process (with pure CA gradually releasing over time), pure CMCSMA hydrogel was used in this study. Time-sweep oscillatory tests were conducted to monitor the changes in the G′ and G″ of the CMCSMA hydrogel, allowing the determination of its gelation time. As shown in Figure 4a, the CMCSMA hydrogel prepolymer solution rapidly formed a hydrogel upon UV irradiation. The short gelation time (15.8 ± 1.5 s) of the hydrogel (Figure 4b) indicates high preparation efficiency, which is advantageous for practical applications. Furthermore, the final G′ and G″ of the CMCSMA hydrogel were measured to be 1099.1 ± 17.7 Pa and 36.1 ± 3.8 Pa, respectively (Figure 4c,d). These results demonstrate that the hydrogel can maintain excellent shape retention and structural stability under external forces.

**Figure 5:**
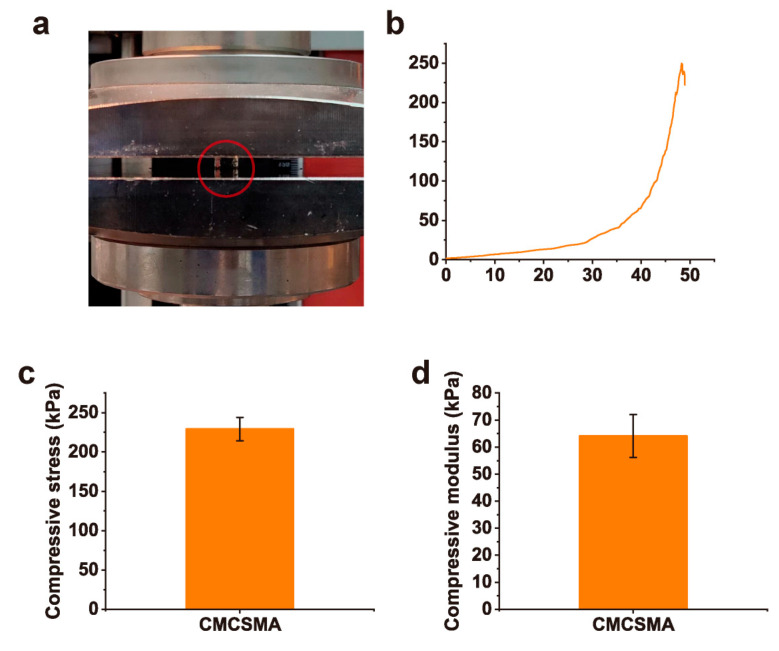
(**a**) Macroscopic image of the compression test of CMCSMA hydrogel; (**b**) stress/strain curve of CMCSMA hydrogel under compression; (**c**) compressive strength of CMCSMA hydrogel; (**d**) compressive modulus of CMCSMA hydrogel.

Uniaxial compression testing was performed to evaluate the mechanical characteristics of the CMCSMA hydrogel (Figure 5a). The resultant compressive stress/strain profile (Figure 5b) demonstrates the hydrogel’s structural response to applied deformation, revealing key parameters such as compressive modulus and yield strength. Additionally, the compressive stress and compressive modulus of the CMCSMA hydrogel were measured to be 229.2 ± 14.8 kPa and 64.1 ± 7.9 kPa, respectively (Figure 5c,d). The compressive stress was higher than other CMCS hydrogels (99.9–130.6 kPa) [39]. The hydrogel has mechanical rigidity and resistance to deformation when coated on the stent. Such characteristics enable the hydrogel to effectively withstand the mechanical forces and tissue activities within the pancreatic duct, ensuring its structural durability and stability under physiological conditions.

### 2.5. Swelling and In Vitro Degradation of CMCSMA Hydrogel Coating

**Figure 6:**
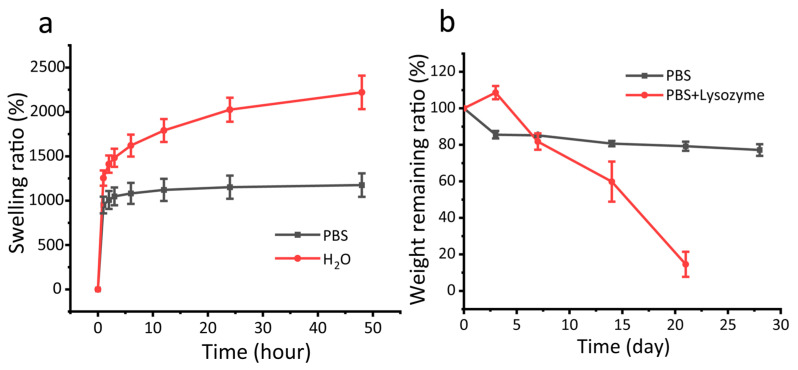
(**a**) The swelling kinetics curves of CMCSMA hydrogel-coated pancreatic duct stent in PBS and H_2_O; (**b**) the degradation kinetics curves of CMCSMA hydrogel-coated pancreatic duct stent in PBS and PBS + lysozyme solution.

As a drug delivery carrier, the swelling properties and degradability of the CMCSMA hydrogel coating play a critical role in facilitating controlled drug release. The swelling kinetics of the CMCSMA hydrogel coating were systematically evaluated in two distinct media: PBS and deionized water. As illustrated in Figure 6a, the hydrogel exhibited markedly different swelling behaviors, with a swelling ratio of 1093.2 ± 22.8% in PBS after 1 h, significantly lower than the 1423.9 ± 54.9% observed in deionized water under identical conditions. The higher swelling of hydrogels in pure water compared to PBS is primarily attributed to osmotic effects and charge shielding [40]. In pure water, the large osmotic pressure difference between the hydrogel network and the surrounding medium drives extensive water penetration to balance the chemical potential, resulting in pronounced swelling. The ionic components of PBS (Na^+^, HPO_4_^2−^) diminish CMCS hydrogel swelling through two mechanisms, osmotic gradient reduction and charge screening of carboxyl groups, which together neutralize electrostatic repulsion and induce network contraction. This indicates that under physiological pH conditions, the hydrogel exhibits a reduced swelling ratio during the initial swelling phase, which is beneficial for slowing the release of therapeutic drugs and decreases the risk of burst release in hydrogel systems. Furthermore, after 24 h of swelling, the swelling ratio of the CMCSMA hydrogel in PBS reached 1315.3 ± 20.0%, which remained lower than that in deionized water (1727.0 ± 107.2%). These swelling characteristics of the hydrogel coating help prevent excessive pressure on the pancreatic duct, mechanical damage, and internal obstruction, thereby ensuring the safety and efficacy of the hydrogel during in vivo applications [20].

The in vitro degradation behavior of the CMCSMA hydrogel coating was studied in PBS and PBS with lysozyme. The hydrogel demonstrated high structural stability in PBS, with a remaining weight ratio of 77.2 ± 3.2% after 28 days (Figure 6b), indicating slow hydrolytic degradation. For the in vitro degradation of CMCSMA hydrogel in PBS with lysozyme, it was observed that the hydrogel degraded slowly in the early stage (81.8 ± 4.5% residual weight at the 7th day), suggesting initial enzyme resistance. After degradation for 14 and 21 days, the remaining weight ratios of the CMCSMA hydrogel were 59.9 ± 11.0% and 14.6 ± 6.9%, respectively, indicating that the hydrogel degrades rapidly in the later stage with the lysozyme. The degradation curve aligns with therapeutic needs: the early phase maintains hydrogel coating integrity for sustained drug delivery. The degradation at the late phase ensures timely clearance post therapy. Therefore, CMCSMA hydrogel exhibits tunable degradation kinetics in enzyme-rich environments, making it a promising candidate for pancreatic duct calculi litholysis.

### 2.6. Hemolysis Assay and Cytocompatibility of CMCSMA Hydrogel Coating

**Figure 7:**
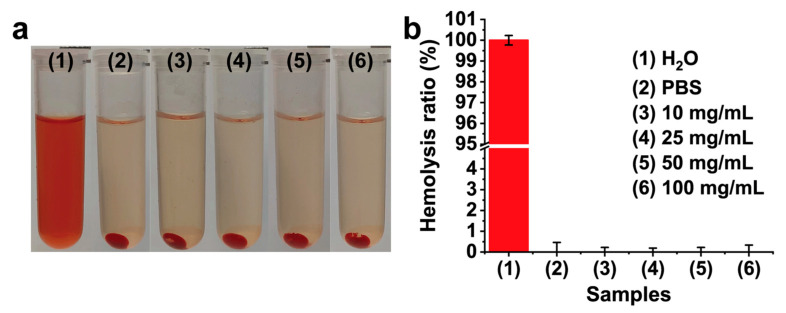
(**a**) Macroscopic images of hemolysis assay; (**b**) hemolysis ratio of different groups.

To eliminate potential interference from the released CA on cells and considering the proven biosafety of the stent, pure CMCSMA hydrogel was used in the hemolysis assay and cytocompatibility studies. The blood compatibility of the CMCSMA hydrogel coating was assessed through an in vitro hemolysis assay. Figure 7a presents macroscopic comparisons of hemolytic activity among the experimental groups following 2 h of incubation including the positive control group, the negative control group, and the CMCSMA hydrogel group. The supernatant of the positive control group exhibited a bright red color, indicating complete hemolysis, while the supernatant of the CMCSMA hydrogel group appeared similar in color to that of the negative control group, suggesting minimal hemolysis. The absorbance of the supernatant was measured, and the hemolysis ratio was calculated to quantitatively evaluate the hemolytic activity of the CMCSMA hydrogel coating. As shown in Figure 7b, no significant hemolytic reaction was observed in the hydrogel groups with varying concentrations of CMCSMA (10, 25, 50, and 100 mg/mL), confirming that the erythrocyte remained intact. A hemolysis rate of less than 5% is generally considered the threshold for non-significant hemolysis [41,42]. The hemolysis ratios of all the experimental groups are below 1%, confirming the non-hemolytic nature of CMCSMA hydrogel.

**Figure 8:**
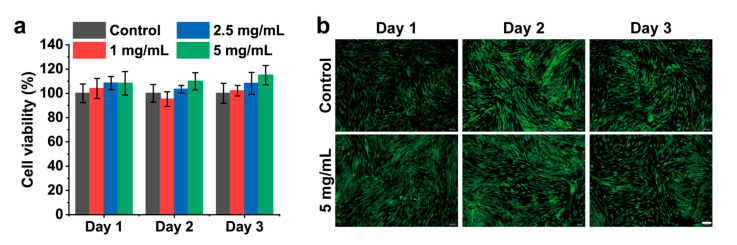
(**a**) Viability of HFB cells after 1, 2, and 3 days of treatment with DMEM and CMCSMA hydrogel leachates at varying concentrations (1, 2.5, and 5 mg/mL); (**b**) live/dead staining fluorescence images of HFB cells after 1, 2, and 3 days of treatment with the CMCSMA hydrogel leachates (5 mg/mL) (scale bar: 100 μm).

HFB cells were employed as an in vitro model system to assess the cytocompatibility of the CMCSMA hydrogel coating through comprehensive cytotoxicity evaluation. As demonstrated by the CCK-8 assay results, HFB cells exhibited unaffected viability after being incubated with hydrogel leachates at concentrations of 1, 2.5, and 5 mg/mL for 1, 2, and 3 days (Figure 8a). Furthermore, the live/dead cell staining results confirmed that the CMCSMA hydrogels exhibited no significant cytotoxicity to HFB cells (Figure 8b). After treatment with the hydrogel leachate at a concentration of 5 mg/mL, HFB cells maintained normal growth and retained their characteristic fusiform morphology. Similar to the control group, the hydrogel-treated group showed a high proportion of viable cells, as indicated by extensive green fluorescence, with only minimal red fluorescence corresponding to a small number of dead cells. The cell viability of the hydrogel leachates remained above 85% in all groups, indicating that the hydrogels had no significant cytotoxicity. These findings demonstrate that the CMCSMA hydrogel possesses excellent cytocompatibility, making it a promising candidate for in vivo implantation and therapeutic applications.

### 2.7. In Vitro Release of CA and In Vitro Litholytic Effect of CMCSMA Hydrogel-Coated Pancreatic Duct Stent

**Figure 9:**
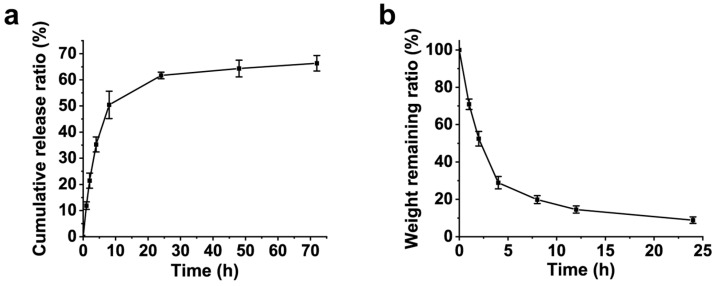
(**a**) Cumulative release curve of CA; (**b**) dissolution curve of pancreatic duct calculi.

Water-soluble drugs can be efficiently released from the hydrogel matrix, a process that usually involves swelling and swelling diffusion [43]. As illustrated in Figure 9a, the cumulative release ratios of CA at 1, 2, 4, and 8 h were 11.9 ± 1.4%, 21.4 ± 2.9%, 35.2 ± 2.9%, and 50.4 ± 5.2%, respectively. The rapid release rate of CA within the first 8 h can be attributed to the hydrogel’s porous structure, which facilitates efficient water absorption and rapid swelling. The cumulative release ratios of CA after 24, 48, and 72 h were 61.7 ± 1.3%, 64.3 ± 3.2%, and 66.3 ± 3.0%, respectively, indicating that the release rate stabilizes after 24 h. These results demonstrate that the prepared CMCSMA hydrogel-coated pancreatic duct stent has significant potential for applications in drug delivery and controlled release systems.

To evaluate the litholytic efficacy of the CMCSMA-CA hydrogel, in vitro dissolution studies were conducted. Figure 9b demonstrates a time-dependent reduction in pancreatic calculi mass, the weight remaining ratios of pancreatic calculi at 1, 2, 4, 8, 12, and 24 h were 70.9 ± 2.8%, 52.4 ± 3.9%, 28.9 ± 3.3%, 19.9 ± 2.1%, 14.6 ± 2.0%, and 8.9 ± 1.8%, respectively, confirming the hydrogel’s potent calculi-dissolving capacity. The rapid dissolution of pancreatic calculi when incubated with the CMCSMA-CA hydrogel-coated stent suggests that the hydrogel-coated stent exhibits a remarkable litholytic effect. This finding indicates that the CMCSMA-CA hydrogel-coated pancreatic duct stent holds promise for further development and application in in vivo pancreatic duct litholysis therapy.

## 3. Conclusions

In this study, a novel CA-loaded CMCSMA hydrogel was successfully synthesized and in situ coated onto a PDA-modified plastic pancreatic duct stent to create a multifunctional drug-eluting stent for the treatment of pancreatic calculi. The CMCSMA hydrogel demonstrated a highly porous architecture conducive to drug loading, as well as excellent rheological and mechanical properties. Hemolysis and cytocompatibility assays confirmed its biosafety, while in vitro release experiments revealed a sustained and controlled release profile of CA over 72 h. The CA-loaded hydrogel-coated stent achieved a calculi dissolution rate exceeding 90% within 24 h, affirming its significant litholytic efficacy. Therefore, compared to naked stents, this hydrogel-coated stent loaded with drugs will ensure the efficient removal of calculi after ERCP combined with ESWL. The dual functionality of mechanical support and targeted drug release positions the CMCSMA-CA hydrogel-coated stent as a promising candidate for advanced endoscopic interventions in pancreatic duct litholysis. In the in vitro degradation experiment, the CMCSMA hydrogel coating demonstrated a slow initial degradation rate followed by accelerated breakdown in the later stages, which enables controlled drug release and ensures complete hydrogel clearance without requiring secondary removal procedures. The findings highlight the promising clinical applicability of this novel drug-eluting stent. Future in vivo evaluations and clinical translation studies will be essential to validate its efficacy and optimize its application in chronic pancreatitis management.

## 4. Materials and Methods

### 4.1. Materials

CMCS (Mw: 100,000–200,000, degree of substitution ≥ 80%) was purchased from Macklin Inc. (Shanghai, China) Tris(hydroxymethyl)aminomethane Hydrochloride (Tris-HCl), citric acid monohydrate, sodium hydroxide, and sodium chloride were purchased from Sinopharm Chemical Reagent Co., Ltd. (Shanghai, China). PBS, MA, dopamine hydrochloride, lysozyme from egg white (extra-pure grade, ≥20,000 U/mg) and 2-Hydroxy-4′-(2-hydroxyethoxy)-2-methylpropiophenone (photoinitiator I2959) were supplied by Aladdin Scientific Corp. (Shanghai, China). The pancreatic duct stents (SPSOF-5-10, batch number C1783145) were obtained from Cook Ireland Limited (Limerick, Ireland). The pancreatic calculi were supplied by Changhai Hospital, Naval Military Medical University. The Cell Counting Kit (CCK-8, CK04, Lot. VG533) was obtained from Dojindo Laboratories (Kumamoto, Japan). The Calcein/PI Live/Dead Viability/Cytotoxicity Assay Kit (C2015M, Lot. 121522230328) was purchased from Beyotime Biotech. Inc. (Shanghai, China).

### 4.2. Preparation and Characterization of CMCSMA

The preparation and dialysis purification of CMCSMA were based on a previous study [44]. First, 10 g of CMCS was completely dissolved in 400 mL of deionized water under constant stirring in a 50 °C water bath. Subsequently, 1 mL of MA was added at a controlled rate of 0.5 mL/min. The resulting mixture was continuously stirred for 4 h at 50 °C to ensure complete reaction. Next, the resulting mixture was subjected to dialysis purification using a 3.5 kDa molecular weight cutoff (MWCO) membrane against deionized water for 96 h to yield purified CMCSMA. The purified CMCSMA was then freeze-dried for subsequent use. The successful synthesis of CMCSMA was verified through ^1^H NMR spectroscopy (NMR, Bruker 400 M, Fällanden, Switzerland).

### 4.3. Preparation and Characterization of the Hydrogel-Coated Stents

The PDA modification of the stent was based on previous research [45]. First, the pancreatic duct stent was cleaned ultrasonically with ethanol and deionized water for 20 min separately. Then, 10 mM Tris-HCl buffer solution was prepared, and its pH was adjusted to 8.5 using NaOH solution. After that, 160 mg of DA powder was added to 80 mL of Tris-HCl buffer solution to prepare the DA solution. The cleaned pancreatic duct stent was placed in the DA solution at room temperature and stirred continuously for 66 h. The stent was taken out and rinsed thoroughly with deionized water to remove unpolymerized DA. Then, it was dried in an oven to obtain a PDA-modified pancreatic duct stent.

CMCSMA was dissolved in deionized water to create a 5.5% *w*/*v* CMCSMA solution. Then, Irgacure 2959 was added to the CMCSMA solution at a concentration of 0.5% *w*/*v* to prepare a hydrogel precursor. The PDA-modified pancreatic duct stent was then placed in this hydrogel precursor solution and exposed to ultraviolet light (365 nm wavelength, 5 W power) for 1 min to form CMCSMA hydrogel in situ and a hydrogel-coated stent was obtained. The hydrogel-coated stent was frozen at −20 °C overnight and subsequently freeze-dried for 3 days. The frozen CMCSMA hydrogel coating samples were carefully sectioned with scissors to expose the cross-section, mounted on a sample stage using conductive adhesive, and sputter-coated with gold. The cross-sectional microscopic morphology of the lyophilized hydrogel was observed using a scanning electron microscope (SEM, Tescan Mira4, Brno-Kohoutovice, Czech Republic). For the preparation of the CA-loaded hydrogel-coated pancreatic duct stent, CA was incorporated into the hydrogel precursor solution at a concentration of 50% *w*/*v*. The same procedure was followed to coat the stent, resulting in the CMCSMA-CA hydrogel-coated pancreatic duct stent. This CMCSMA-CA hydrogel-coated pancreatic duct stent was also lyophilized, and its internal microscopic structure was observed using SEM.

### 4.4. Rheological Properties and Mechanical Properties of the Hydrogel

The rheological properties of CMCSMA hydrogel were evaluated at 37 °C using a rotational rheometer (MARS III Haake, Zwick Roell (Shanghai, China), *n* = 3). A 250 μL of hydrogel prepolymer solution was carefully loaded between parallel plates (P20 TiL, diameter: 20 mm, gap: 1 mm). Time-sweep oscillatory tests were performed under controlled conditions, with a constant strain of 1% and a constant frequency of 1 Hz. The gelation time was identified as the point at which the storage modulus (G′) surpassed the loss modulus (G″).

The mechanical properties of CMCSMA hydrogel were assessed using a universal material testing machine (Zwick Roell Z2.5 TH with a 2.5 kN sensor) (*n* = 3). Hydrogel samples were molded into cylindrical shapes (diameter: 8 mm, height: 4 mm) for testing. The cylindrical hydrogel samples underwent uniaxial compression testing at a controlled strain rate of 1 mm/min, producing characteristic stress/strain profiles for mechanical analysis. The compressive strength of the hydrogel was defined as the stress at the rupture point. The compressive modulus was derived from the linear region of the stress/strain curve, specifically within the 10–20% strain range, using linear regression analysis.

### 4.5. Swelling and Degradation Tests

To study the real-world swelling and degradation behaviors, CMCSMA hydrogel-coated stents were used. For the swelling behavior evaluation (*n* = 3), the CMCSMA hydrogel-coated pancreatic duct stents were lyophilized and weighed to obtain the dry weight (*W_d_*). The stents were then immersed in PBS and deionized water, and incubated at 37 °C. At predetermined time intervals (1, 2, 3, 6, 12, and 24 h), the stents were carefully removed from the PBS and deionized water, and excess surface moisture was gently blotted using filter paper. The wet weight (*W_s_*) of each stent was measured immediately to calculate the swelling ratio according to Formula (1).

(1) $$Swelling\;ratio\left(\%\right)=\frac{W_{s}-W_{d}}{W_{d}}\times 100\%$$

For the degradation test (*n* = 3), the CMCSMA hydrogel samples were first lyophilized, and their initial dry weights were recorded as *W*_0_. Then, the CMCSMA hydrogel was individually immersed in two media, (1) PBS and (2) PBS containing lysozyme, and incubated at a constant temperature of 37 °C for predetermined time intervals (3, 7, 14, 21, and 28 days). The solution was replaced daily to maintain consistent conditions. At predetermined intervals, the residual hydrogel specimens were meticulously collected, rigorously washed with deionized water to remove ionic contaminants, and finally freeze-dried to complete the purification process. The dry weights of the degraded samples were recorded as *W_t_* to calculate the residual weight ratio according to Formula (2).

(2) $$Weight\;remaining\;ratio\;\left(\%\right)=\frac{W_{t}}{W_{0}}\times 100\%$$

### 4.6. Hemolysis Assay

The blood compatibility of the CMCSMA hydrogel coating was evaluated through a hemolysis assay (*n* = 3). Fresh whole blood was collected from KM mice. Animal studies were approved by the Animal Ethics Committee of Ganzhou People’s Hospital (No. PJD2025-007-01), and all operations followed the Regulations on the Management of Laboratory Animals (SYXK(Gan)2023-0006). The blood was centrifuged at 5000 rpm for 5 min and washed 5 times with PBS to isolate red blood cells. The obtained red blood cells were resuspended in 50 mL of PBS to prepare a red blood cell suspension. For the assay, PBS containing varying concentrations of CMCSMA hydrogel (10, 25, 50, and 100 mg/mL) was mixed with the red blood cell suspension and incubated at 37 °C in a constant temperature incubator. A mixture of deionized water and red blood cell suspension served as the positive control, while a mixture of PBS and red blood cell suspension was used as the negative control. After 2 h of incubation, 2 mL of each mixture was collected and centrifuged at 3000 rpm for 5 min to obtain the supernatant. The absorbance of the supernatant was measured at 541 nm by a UV-visible spectrophotometer (UV1800PC, Jinghua, Shanghai, China) to calculate the hemolysis ratio using Formula (3):

(3) $$Hemolysis\;ratio\;\left(\%\right)\;=\;\frac{A_{h}-A_{n}}{A_{p}-A_{n}}\times 100\%$$

where *A_h_* represents the absorbance of the supernatant of the hydrogel group, *A_p_* represents the absorbance of the supernatant of the positive control group, and *A_n_* represents the absorbance of the supernatant of the negative control group.

### 4.7. In Vitro Cytocompatibility

The cytocompatibility of CMCSMA hydrogel coating was assessed both quantitatively and qualitatively using the CCK-8 assay and live/dead cell staining, respectively, with human fibroblast (HFB) cells (*n* = 3). The CMCSMA hydrogel was first lyophilized and sterilized for 12 h. Hydrogel leachates were prepared by incubating the sterilized hydrogel in Dulbecco’s Modified Eagle’s Medium (DMEM) supplemented with 10% fetal bovine serum (FBS), 100 U/mL penicillin, and 100 mg/mL streptomycin at varying concentrations (1, 2.5, and 5 mg/mL) for 24 h at 37 °C. HFB cells were seeded in 96-well cell culture plates at a density of 1 × 10^4^ cells per well and incubated for 24 h at 37 °C. After removing the original culture medium, the hydrogel leachates were added to the respective wells. For the control group, HFB cells were cultured in DMEM without hydrogel leachates. Cell viability was evaluated using the CCK-8 assay and live/dead cell staining assay after 1, 2, and 3 days of incubation at 37 °C. Cell morphology was observed and imaged using an inverted fluorescence microscope (Olympus, Olympus Corporation of Japan, Tokyo, Japan, magnification: 20×).

### 4.8. Citric Acid Loading Content and Release Behavior Study

The prepared CMCSMA-CA hydrogels (containing 50% *w*/*v* CA) were placed in dialysis bags (MWCO: 3500 Da). The dialysis bags were then immersed in 10 mL of deionized water and incubated in an oscillating culture incubator (90 rpm, 37 °C, *n* = 3). At predetermined time intervals (1, 2, 4, 8, 24, 48, and 72 h), 1 mL of the CA-containing solution was collected and replaced with an equal volume of fresh deionized water to maintain a constant volume. The released CA in the collected samples was quantified using spectrophotometry. Specific operations are as follows: the above CA-containing solution sample was mixed with 4 mL of 0.1 M Fe(NO_3_)_3_ solution and 1 mL of 0.1 M HNO_3_ solution in a 25 mL colorimetric tube. The mixture was then diluted to a final volume of 25 mL with deionized water. The absorbance of the resulting solution was measured at 490 nm using a UV-visible spectrophotometer (UV1800PC, Jinghua, Shanghai, China). The CA concentration in each sample was determined based on a pre-established standard curve of CA. Finally, the cumulative release rate of CA was calculated, and the release profile was plotted using Origin software (Origin 2024).

### 4.9. In Vitro Calculi Dissolving Effect

The pancreatic calculi, obtained from Changhai Hospital, were co-incubated with the CMCSMA-CA hydrogel-coated stents (containing 50% *w*/*v* CA) in 10 mL of deionized water. The mixture was placed in an oscillating culture incubator at 37 °C (90 rpm, *n* = 3). At predetermined time intervals (1, 2, 4, 8, 12, and 24 h), the pancreatic calculi were carefully removed from the solution, and excess moisture was absorbed using filter paper. The calculi were then dried and weighed to determine their residual weight and calculate the weight remaining ratio using Equation (4).

(4) $$Weight\;remaining\;ratio\;\left(\%\right)=\frac{W_{t}}{W_{0}}\times 100\%$$

where *W_t_* represents the calculi weight at a certain point, and *W*_0_ represents the initial calculi weight.

## Data Availability

The data presented in this study are available on request from the corresponding author.
