# Correction: Li et al. CMCSMA-Citric Acid Hydrogel-Coated Pancreatic Duct Stent Used for Pancreatic Calculi. *Gels* 2025, *11*, 651

**DOI:** 10.3390/gels11100820

**Published:** 2025-10-13

**Authors:** Jing Li, Jiahao Yang, Shige Wang

**Affiliations:** 1 School of Materials and Chemistry, University of Shanghai for Science and Technology, No. 334 Jungong Road, Shanghai 200093, China; lijing@usst.edu.cn (J.L.); y2952847512@163.com (J.Y.); 2 Public Experiment Center, University of Shanghai for Science and Technology, No. 334 Jungong Road, Shanghai 200093, China

The authors would like to make the following correction to [1]: in the original publication, Ref. [36] was not cited. The citation has now been inserted in the caption of Figure 1.

The original Refs. [36–44] have now been updated as Refs. [37–45] in the reference citations of the main text; we have changed the order of references and have adjusted accordingly. The authors state that the scientific conclusions are unaffected. This correction was approved by the Academic Editor. The original publication has also been updated.

**References List**

We added reference [36], and modified the order of the references from [37] to [45]:

36. Yang, J.; Wang, T.; Zhang, L.; Fan, P.; Zhao, J.; Zheng, X.; Lai, Y.; Liu, H.; Wang, S. Injectable hemostatic hydrogel adhesive with antioxidant, antibacterial and procoagulant properties for hemorrhage wound management. *J. Colloid Interface Sci.* **2024**, *673*, 395–410.
37. Obiweluozor, F.O.; Maharjan, B.; Emechebe, A.G.; Park, C.H.; Kim, C.S. Mussel-inspired elastic interpenetrated network hydrogel as an alternative for anti-thrombotic stent coating membrane. *Chem. Eng. J.* **2018**, *347*, 932–943.
38. Liu, M.; Zeng, G.; Wang, K.; Wan, Q.; Tao, L.; Zhang, X.; Wei, Y. Recent developments in polydopamine: An emerging soft matter for surface modification and biomedical applications. *Nanoscale* **2016**, *8*, 16819–16840.
39. Chen, M.; Tan, H.; Xu, W.; Wang, Z.; Zhang, J.; Li, S.; Zhou, T.; Li, J.; Niu, X. A self-healing, magnetic and injectable biopolymer hydrogel generated by dual cross-linking for drug delivery and bone repair. *Acta Biomater.* **2022**, *153*, 159–177.
40. De Piano, R.; Caccavo, D.; Barba, A.A.; Lamberti, G. Swelling Behavior of Anionic Hydrogels: Experiments and Modeling. *Gels* **2024**, *10*, 813.
41. Wang, Y.; Yang, M.; Zhao, Z. Facile fabrication of self-healing, injectable and antimicrobial cationic guar gum hydrogel dressings driven by hydrogen bonds. *Carbohydr. Polym.* **2023**, *310*, 120723.
42. Pansuriya, R.; Patel, T.; Singh, K.; Al Ghamdi, A.; Kasoju, N.; Kumar, A.; Kailasa, S.K.; Malek, N.I. Self-healable, stimu-li-responsive bio-ionic liquid and sodium alginate conjugated hydrogel with tunable Injectability and mechanical properties for the treatment of breast cancer. *Int. J. Biol. Macromol.* **2024**, *277*, 134112.
43. Van Tienderen, G.S.; Berthel, M.; Yue, Z.; Cook, M.; Liu, X.; Beirne, S.; Wallace, G.G. Advanced fabrication approaches to controlled delivery systems for epilepsy treatment. *Expert. Opin. Drug Del.* **2018**, *15*, 915–925.
44. Risangud, N.; Lertwimol, T.; Sitthisang, S.; Wongvitvichot, W.; Uppanan, P.; Tanodekaew, S. The preparation of 3D-printed self-healing hydrogels composed of carboxymethyl chitosan and oxidized dextran via stereolithography for biomedical ap-plications. *Int. J. Biol. Macromol.* **2025**, *292*, 139251.
45. Lee, H.; Dellatore, S.M.; Miller, W.M.; Messersmith, P.B. Mussel-Inspired Surface Chemistry for Multifunctional Coatings. *Science* **2007**, *318*, 426–430.

## Figures and Tables

**Figure 1:**
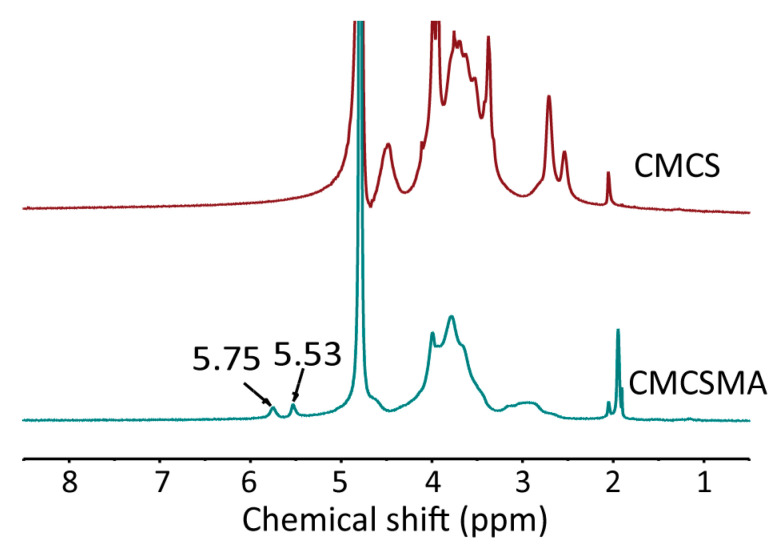
The ^1^H spectra of CMCS and CMCSMA [36].
